# Future career plans of medical students and the COVID‐19 pandemic: Time to recover?

**DOI:** 10.1002/jgf2.591

**Published:** 2022-11-07

**Authors:** Houman Goudarzi, Masahiro Onozawa, Makoto Takahashi

**Affiliations:** ^1^ Faculty of Medicine and Graduate School of Medicine, Center for Medical Education and International Relations Hokkaido University Sapporo Japan; ^2^ Clinical Training Center Hokkaido University Hospital Sapporo Japan

## Abstract

In 2019 and before the COVID‐19 pandemic, about 68% of our medical students in Japan wished to engage in academic activities abroad. However, in 2020 and during the pandemic, this percentage fell to 35%. We found a significant increase in the number of students wishing to go abroad for studies/training in 2021 than in 2020, taking the percentage to the prepandemic level in 2019.

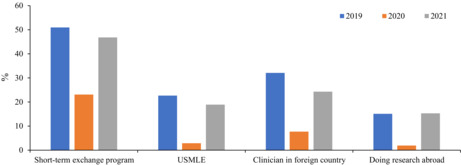

To The Editor,

The coronavirus disease 2019 (COVID‐19) pandemic has impacted medical education tremendously.^1^ The unprecedented disruption in medical schools, hospitals, and healthcare systems globally is expected to make it difficult for medical students to plan their careers, especially considering international medical education and outbound mobility. To address the pandemic's potential effect on medical students' plans to study or train abroad, we recently assessed the pandemic's effect on career plans of medical students before and during the pandemic (2016–19 and 2020) by conducting a questionnaire‐based survey (*n* = 534).^2^

Between 2016 and 2019, we observed an increasing trend of outbound mobility—participation in short‐term international exchange programs, taking the United States Medical Licensing Examination (USMLE), clinical training, and research abroad—among our second‐year medical students.^2^ In 2019, about 68% of the students wished to engage in at least one of the abovementioned academic activities. However, in 2020 and during the pandemic, this percentage fell to 35% for these activities: participation in short‐term exchange programs (−27.9%), taking the USMLE (−19.8%), clinical training (−24.5%), and research abroad (−13.2%) (Figure 1). Thus, the pandemic adversely and significantly influenced our medical students' plans involving outbound mobility.

**FIGURE 1:**
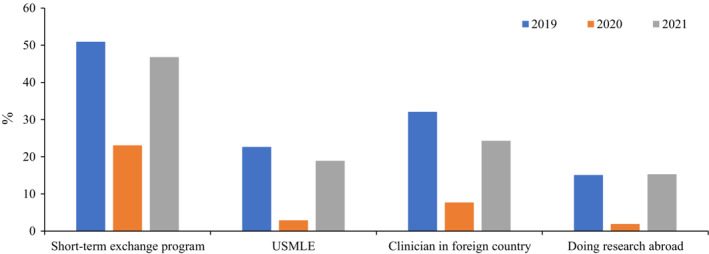
Future career plans of Japanese medical students between 2019 and 2021.

Because of our concern regarding the pandemic's adverse effect on the trajectory of medical education and student mobility, we continued monitoring our second‐year students' career plans in 2021. Interestingly, we found a significant increase in the number of students wanting to go abroad for studies/training in 2021 than in 2020, taking the percentage for the assessed plans close to their prepandemic level in 2019: short‐term exchange programs (−4.1%), taking the USMLE (−3.7%), clinical training (−7.7%), and research abroad (+0.3%) (Figure 1). Of note, the 2021 results showed a higher percentage of students who wished to participate in international medical education and develop their careers abroad than those in the period from 2016 to 2018.

A noticeable recovery in the number of students wanting to be trained/conduct research abroad in 2021 compared with 2020 could be because of several reasons, one being vaccination progress. Also, adopting recovery programs in most countries, efforts made by universities in response and preparedness toward the COVID‐19 pandemic, contemporary immigration and visa support, re‐opening international borders, easing border measures, etc., could be other influential factors. Therefore, we believe that such a rapid recovery is promising and needs replication and more longitudinal surveys in the future. We hope to observe a more encouraging trend in the future as a positive of the pandemic, emphasizing the importance of preparedness in academia, healthcare systems, and society to prevent or minimize the effect of possible future pandemics on medical education.

## FUNDING INFORMATION

This article has not had any external funding.

## CONFLICT OF INTEREST

The authors do not have any conflict of interest to declare. The authors alone are responsible for the content and writing the article.

## ETHICAL APPROVAL

All participants gave written informed consent. Ethical approval for this study was obtained from the Institutional Review Board of the Faculty of Medicine and Graduate School of Medicine, Hokkaido University (20–040).
